# The Role of Platelet-Derived ADP and ATP in Promoting Pancreatic Cancer Cell Survival and Gemcitabine Resistance

**DOI:** 10.3390/cancers9100142

**Published:** 2017-10-24

**Authors:** Omar Elaskalani, Marco Falasca, Niamh Moran, Michael C. Berndt, Pat Metharom

**Affiliations:** 1 Platelet Research Laboratory, School of Biomedical Sciences, Curtin Health and Innovation Research Institute, Faculty of Health Sciences, Curtin University, Bentley, WA 6102, Australia; omar.elaskalani@postgrad.curtin.edu.au; 2 Metabolic Signalling Group, School of Biomedical Sciences, Curtin Health Innovation Research Institute, Curtin University, Bentley, WA 6102, Australia; marco.falasca@curtin.edu.au; 3 Molecular and Cellular Therapeutics, Royal College of Surgeons in Ireland, Dublin 2, Ireland; nmoran@rcsi.ie; 4 Faculty of Health Sciences, Curtin University, Bentley, WA 6102, Australia; m.berndt@curtin.edu.au; 5 Platelet Research Laboratory, Curtin Health and Innovation Research Institute, Faculty of Health Sciences, Curtin University, Bentley, WA 6102, Australia

**Keywords:** platelets, ADP, ATP, pancreatic cancer, gemcitabine

## Abstract

Platelets have been demonstrated to be vital in cancer epithelial-mesenchymal transition (EMT), an important step in metastasis. Markers of EMT are associated with chemotherapy resistance. However, the association between the development of chemoresistance, EMT, and the contribution of platelets to the process, is still unclear. Here we report that platelets regulate the expression of (1) human equilibrative nucleoside transporter 1 (hENT1) and (2) cytidine deaminase (CDD), markers of gemcitabine resistance in pancreatic cancer. Human ENT1 (hENT1) is known to enable cellular uptake of gemcitabine while CDD deactivates gemcitabine. Knockdown experiments demonstrate that Slug, a mesenchymal transcriptional factor known to be upregulated during EMT, regulates the expression of hENT1 and CDD. Furthermore, we demonstrate that platelet-derived ADP and ATP regulate Slug and CDD expression in pancreatic cancer cells. Finally, we demonstrate that pancreatic cancer cells express the purinergic receptor P2Y_12_, an ADP receptor found mainly on platelets. Thus ticagrelor, a P2Y_12_ inhibitor, was used to examine the potential therapeutic effect of an ADP receptor antagonist on cancer cells. Our data indicate that ticagrelor negated the survival signals initiated in cancer cells by platelet-derived ADP and ATP. In conclusion, our results demonstrate a novel role of platelets in modulating chemoresistance in pancreatic cancer. Moreover, we propose ADP/ATP receptors as additional potential drug targets for treatment of pancreatic cancer.

## 1. Introduction

Despite remarkable advancements in our understanding of cancer development and progression, pancreatic ductal adenocarcinoma (PDAC) continues to be one of the most aggressive types of cancer, with a mortality rate that has not changed for the last 50 years. In addition to relatively late stage symptoms and high metastasis, chemotherapy resistance contributes significantly to increased mortality in PDAC [1]. Hypercoagulable disorders often characterise people living with PDAC, marked particularly with an increased risk of venous thromboembolism (VTE) [2]. The association of PDAC with thrombotic events thus suggests a close interplay between cancer and platelets, the key player in haemostasis and thrombosis.

Within the blood compartment, tumour cells can form aggregates with platelets to avoid natural killer cell-mediated cytotoxicity [3,4,5]. PDAC cells can induce platelet activation and aggregation [6]. Once activated, platelets release growth factors and angiogenic factors which contribute to cancer progression [7,8,9]. Platelets through direct contact or release of Transforming growth factor beta 1 (TGFβ1) can activate epithelial-mesenchymal transition (EMT) in tumour cells, an important step in cancer metastasis [10,11]. EMT is a phenotypic alteration in cancer cells towards a more metastatic phenotype characterised by elongated shape and advanced ability to invade and migrate to distant organs, associated with a change in the level of several adhesion proteins and transcriptional factors. For example, Slug, Snail, Twist and Zinc finger E-box-binding homeobox 1 (Zeb-1) are described as mesenchymal transcriptional factors. Upregulation of some or all these factors may predispose cells to a mesenchymal phenotype [12]. Recently it has been shown that EMT may play a role in cancer progression other than by promoting metastasis. For example, Snail and Slug have been associated with chemotherapy resistance [13,14]. Moreover, gemcitabine-resistant PDAC cells display an EMT signature [15], while deletion of the EMT program in a pancreatic cancer mouse model enhanced gemcitabine sensitivity [16].

Gemcitabine is a prodrug cytidine analogue that is clinically used to manage PDAC. Like most cytidine analogues, the activity of gemcitabine is dependent on its cellular uptake, drug activation and deactivation rates. Gemcitabine is a hydrophilic drug. Therefore penetration through the lipid bilayer of the plasma membrane is a crucial step in gemcitabine-mediated cytotoxicity. The human nucleoside transporters mediate cellular uptake of gemcitabine. Deoxycytidine kinase mediates the phosphorylation-dependent activation of gemcitabine, while cytidine deaminase (CDD) catalyses gemcitabine deactivation [17,18,19]. Indeed, overexpression of hENT1 was associated with enhanced gemcitabine response while downregulation of CDD reduced gemcitabine resistance [20,21]. CDD expression is relatively high in some organs like liver [22], where most drugs passage on their transit to the target organ. 

In this study, we have explored if platelets can drive gemcitabine resistance in PDAC cells. To investigate this, we exposed cancer cells to platelets or the releasate from aggregated platelets and examined the level of several proteins that are known to impart gemcitabine resistance in PDAC (Slug, CDD, hENT1) and the phosphorylated level of the survival signalling molecules such as Protein kinase B (also known as Akt) and Extracellular signal-regulated kinase (Erk). We also found that the platelet-derived nucleotides, ADP and ATP, are the main soluble mediators that drive gemcitabine resistance, which is completely blocked by ticagrelor, an ADP P_2_Y_12_ receptor antagonist, and that the P_2_Y_12_ receptor antagonist is expressed not only on platelets but also on PDAC cells.

## 2. Results

### 2.1. Platelet Releasate Promotes Proliferation and Survival Signals in PDAC Cells Challenged with Gemcitabine

**Figure 1:**
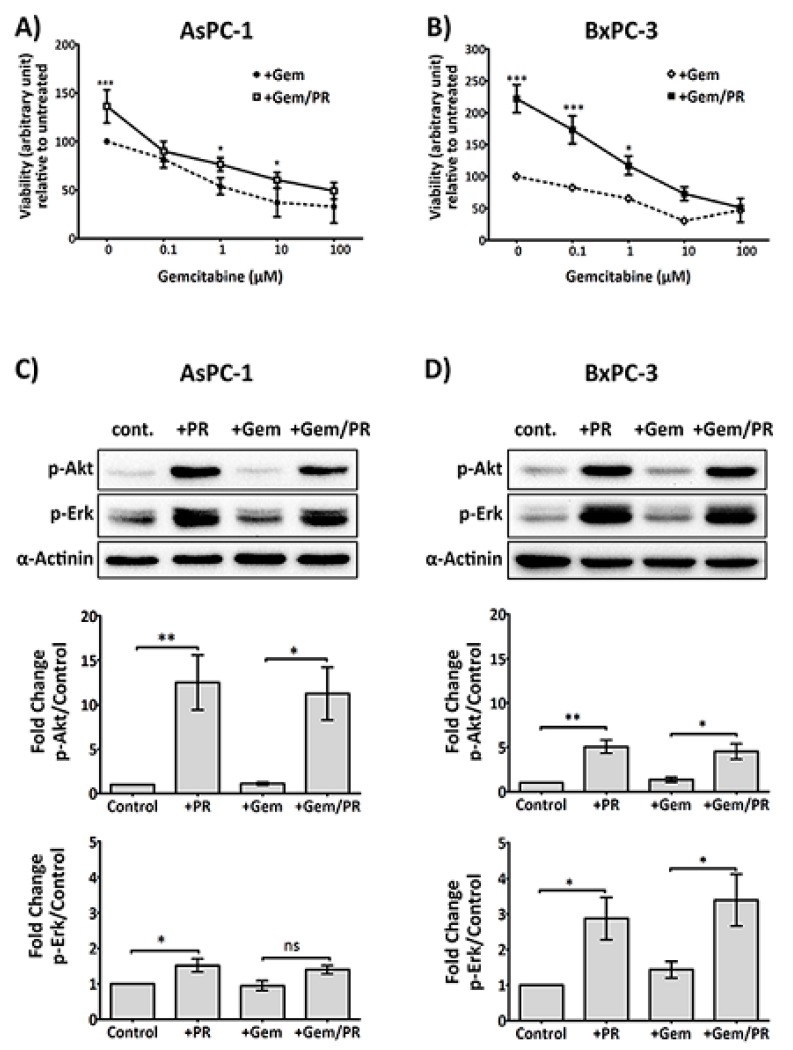
Platelet releasate (PR) promotes pancreatic ductal adenocarcinoma (PDAC) cell survival in the presence of gemcitabine. PDAC cell lines, AsPC-1 (**A**) or BxPC-3 (**B**), were seeded at 5000 cells/well, in a 96-well plate for 24 h, then treated with platelet releasate (PR) ± gemcitabine (0 to 100 µM) for 72 h. PR was prepared from xl-CRP (1 µg/mL)-aggregated platelets (5 × 10^9^ platelets/mL) and used in cell culture at 1:10 dilution, resulting in the final concentration equivalent to the amount of releasate from 5 × 10^8^ platelets/mL. Cell proliferation was quantified by Alamar blue reagent, a non-toxic cell health indicator dye that is converted to fluorescent red colour in living cells. The fluorescence intensity (ex/em 570 nm/610 nm) was quantified by a multi-mode plate reader (PerkinElmer). Statistics were calculated using two-way ANOVA, with *n* = 5 for (**A**) and *n* = 4 for (**B**), *** *p* < 0.0001, * *p* < 0.05. Changes in the phosphorylation status of Protein kinase B (Akt) and Extracellular signal-regulated kinase (Erk) in cancer cells at 2 h were determined following high-dose gemcitabine treatment (25 µM for AsPC-1 and 10 µM for BxPC-3). Briefly, cancer cells were seeded at 3 × 10^5^ cells/well in a 6-well plate for 24 h, serum starved for 6 h, then treated with PR ± gemcitabine for 2 h. Cell lysates were separated by SDS-PAGE and immunoblotted using phospho-specific antibodies for Akt (upper panel) and Erk1/2 (middle panel). Alpha (α)-actinin (lower panel) was used as loading controls for each protein. The expression level of the protein of interest was quantified and normalised to the loading control with automated software Image Lab (version 5.1, BioRad, CA, USA) and GraphPad Prism 5 (GraphPad Software, Inc, CA, USA). The columns represent fold changes in protein expression level compared to vehicle control-treated cells. Data are presented as mean ± SEM. One-way ANOVA with post-hoc Bonferroni’s Multiple Comparison Test was used to examine the significance of the mean, with *n* ≥ 4 for (**C**) and *n* ≥ 3 for (**D**), ** *p* < 0.001, * *p* < 0.05. Abbreviations: Gem—gemcitabine, xl-CRP—cross-linked Collagen-related peptide, PDAC—pancreatic ductal adenocarcinoma, PR—platelet releasate, Akt—Protein kinase B, Erk—Extracellular signal-regulated kinase, SDS-PAGE—sodium dodecyl sulfate polyacrylamide gel electrophoresis, ANOVA—Analysis of variance.

High platelet count is associated with chemotherapy resistance and poor prognosis [23]. It is known that activated platelets release a variety of growth factors that support cancer cell proliferation and survival [24,25,26]. In order to address if platelets also enable PDAC cells to survive the antiproliferative effect of gemcitabine, platelet releasate isolated from collagen-related peptide (CRP)-aggregated platelets was incubated with cancer cells at increasing concentrations of gemcitabine (0–100 µM) for 72 h. As shown in Figure 1A,B, platelet releasate induced a significant increase in proliferation in PDAC cell lines, AsPC-1 and BxPC-3, despite gemcitabine challenge (*n* ≥ 4; AsPC-1, *p* < 0.05 and BxPC-3, *p* < 0.05 at 10 μM and 1 μM gemcitabine, respectively). 

Next, we examined if platelet releasate can initiate survival signals in cancer cells challenged with gemcitabine. AsPC-1 cells are considered gemcitabine-resistant, while BxPC-3 cells are gemcitabine sensitive. Therefore, two different concentrations of gemcitabine were used with the two cell lines. AsPC-1 and BxPC-3 cells were incubated with platelet releasate ± gemcitabine (25 µM and 10 µM, respectively) for 2 h in Roswell Park Memorial Institute (RPMI) serum-free medium. 

As shown in Figure 1C,D, platelet releasate triggered a significant upregulation of phosphorylation of the survival signalling molecules, p-Erk and p-Akt, in both AsPC-1 and BxPC-3 cells, which was unaffected by the presence of gemcitabine. 

### 2.2. Platelet Releasate Induces A Rapid Upregulation of the EMT Transcription Factor, Slug, Independent of the TGFβ/Smad Pathway

Platelets can affect a change in cancer cells by upregulation of relevant transcription factors involved in the EMT process, including Snail, Slug, and Twist [10,27,28]. One such marker, Slug, has recently been implicated in supporting chemotherapy resistance activity in tumour cells [13,14,29]. The main platelet-derived soluble factor that has been suggested to regulate Slug expression is TGFβ1, through the Smad effector pathway [10,30]. However, TGFβ/Smad signalling defects and non-Smad signalling pathways have been observed in several conditions and cell types [31,32,33]. Therefore, we investigated the expression of Slug and TGFβ/Smad signalling in AsPC-1 and BxPC-3 cells stimulated with platelet releasate. 

**Figure 2:**
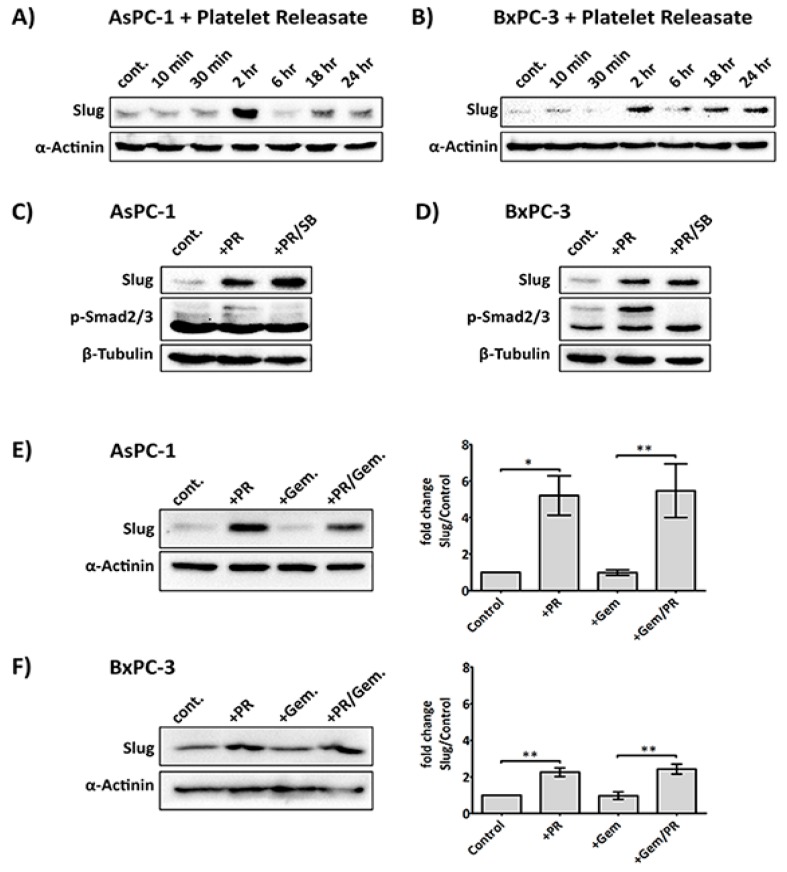
PR induces a rapid upregulation of Slug, an EMT and chemotherapy resistance marker, independent of the TGFβ1/Smad pathway. Representative immunoblots (**A**) and (**B**) show Slug expression in AsPC-1 and BxPC-3 cells after time course treatment with platelet releasate (PR). 2 × 10^5^ cancer cells per well were seeded in a 12-well plate for 24 h, serum starved for 6 h, then PR was added to the culture media (final concentration of PR was equivalent to releasate from 5 × 10^8^ platelets/mL) for 10 min, 30 min, 2 h, 6 h, 18 h and 24 h. Representative immune blots (**C**) and (**D**) show Slug and pSmad2/3 expression in AsPC-1 and BxPC-3 cells after treatment with PR ± 10 µM SB431542 (TGFβ1 receptor inhibitor) or 0.1%DMSO (vehicle control, Sigma-Aldrich, St. Louis, MO, USA) for 2 h. Cell lysates were separated by SDS-PAGE and immunoblotted using specific antibodies for Slug (upper panel), p-Smad2/3 (middle panel) and loading control protein α-actinin or β-tubulin (lower panel). Each of the immunoblots (**A**)–(**D**) is representative of two independent experiments with similar results. Representative immune blots (**E**) and (**F**) and the associated bar graphs show Slug expression in AsPC-1 and BxPC-3 cells after 2 h PR ± gemcitabine treatment. The expression level of the protein of interest was quantified relative to the loading control. The graph columns represent fold changes in protein expression level compared to vehicle-treated cells (*n* = 4 for (**E**) and *n* > 3 for (**F**)). One way ANOVA with post.-hoc Bonferroni’s Multiple Comparison Test was used to examine the significance of the mean (** *p* < 0.001, * *p* < 0.05).

The releasate used in our study was determined to contain approximately 10 ng/mL of TGF-β1 by ELISA (Supplementary Figure S1), which is in line with the previous reported literature [34]. As shown in Figure 2A,B, platelet releasate induced a rapid upregulation of Slug expression after 2 h in both AsPC-1 and BxPC-3. Interestingly, the increase in Slug by platelet releasate was not entirely due to TGFβ1, as Slug level remained upregulated in the presence of a TGFβ1 receptor inhibitor (SB431542 (Tocris Bioscience, Bristol, UK), Figure 2C,D. And the increase of Slug expression by TGFβ1 is much more elevated in BxPC-3 compared to AsPC-1, suggesting the cell lines may have distinct responses to TGFβ1 (Supplementary Figure S2). Additionally, SB431542 reduced the phosphorylation of Smad2/3 in both platelet releasate-stimulated AsPC-1 and BxPC-3 cells. Moreover, platelet releasate was able to sustain Slug upregulation in cancer cells challenged with gemcitabine (Figure 2E,F).

### 2.3. Platelets Modulate hENT1 and CDD in PDAC Cells

**Figure 3:**
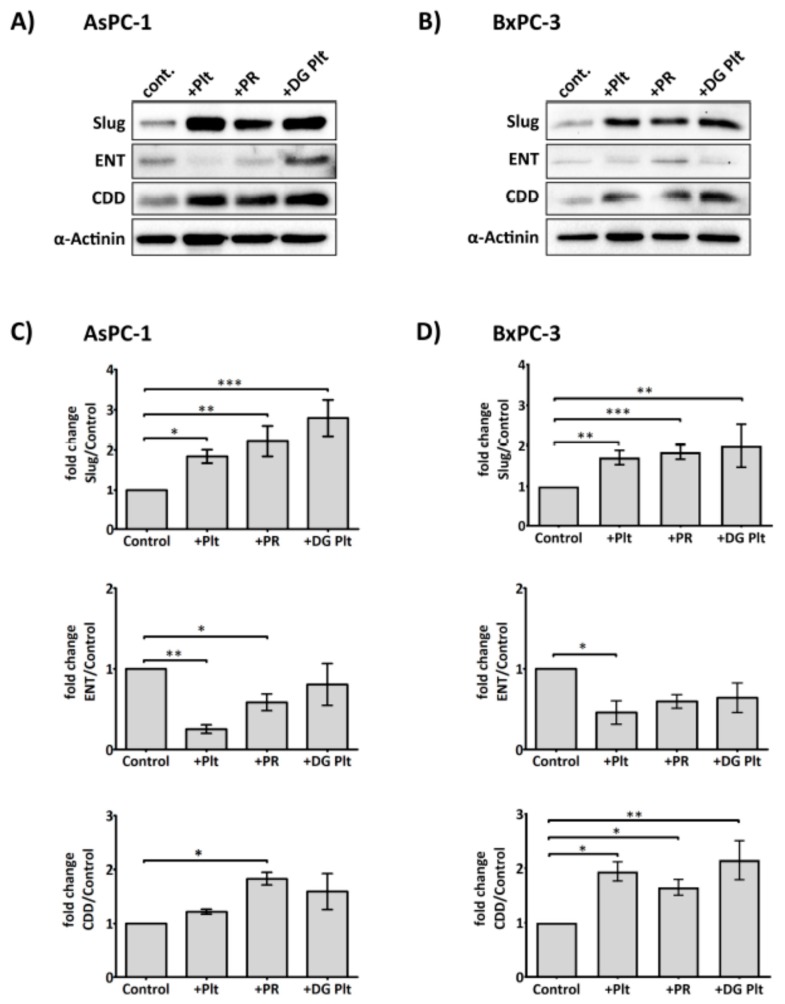
Platelets modulate the expression of Slug, human equilibrative nucleoside transporter 1 (hENT1) and cytidine deaminase (CDD) in PDAC cells. Representative immunoblots (**A**) and (**B**) show the expression of Slug, hENT1 and CDD in AsPC-1 and BxPC-3 after treatment with platelets (Plt), platelet releasate (PR) or degranulated platelets (DG Plt) for 24 h. PR and degranulated platelets (DG Plt) were isolated from activated platelets. Briefly, platelets (1 × 10^9^/mL) were aggregated by incubating with 1 µg/mL CRP for 30 min then the supernatant (i.e., PR) and the pellet (i.e., DG Plt) were separated by centrifugation (5000 *g* for 10 min). The pellet was resuspended in Tyrode’s buffer, using the initial volume. The cancer cells were seeded in a 6-well plate at 3 × 10^5^ per well for 24 h, then incubated with Plt, PR or DG Plt to the final concentration equivalent to 1 × 10^8^ platelets/mL for 24 h in serum-free media. Cell lysates were separated by SDS-PAGE and immunoblotted using specific antibodies for Slug, hENT1, CDD and loading control protein α-actinin. Bar graphs (**C**) and (**D**) show the changes in the expression of Slug, hENT1 and CDD in AsPC-1 and BxPC-3 after different treatments. The expression level of the protein of interest was quantified relative to the loading control and normalised to the negative control group (*n* ≥ 3). Data are presented as mean ± SEM. One way ANOVA with post-hoc Bonferroni’s Multiple Comparison Test was used to examine the significance of the mean. *** *p* < 0.0001, ** *p* < 0.001, * *p* < 0.05.

As platelet releasate could promote cancer cell proliferation and activation of key kinases involved in supporting cell survival, Erk and Akt, despite the presence of gemcitabine, we hypothesised that platelet-derived factors could modulate the cellular metabolism and uptake of gemcitabine to counteract its cytotoxic effects. The influx of gemcitabine depends primarily on the nucleoside transporters [35]. Once inside the cell, gemcitabine can be deactivated by CDD, an enzyme whose increased expression has been implicated in chemoresistance [36,37]. As shown in Figure 3, platelet releasate (equivalent to 1 × 10^8^ platelets/mL) significantly augmented the expression of CDD in both AsPC-1 and BxPC-3 cell lines. The level of hENT1 was decreased by platelet releasate in the two cell lines, with statistically significant reduction observed in AsPC-1. Unstimulated or degranulated platelets, however, were less effective at modulating the expression levels of hENT1 and CDD.

### 2.4. Slug Modulates Expression of CDD and hENT1 in PDAC Cells

**Figure 4:**
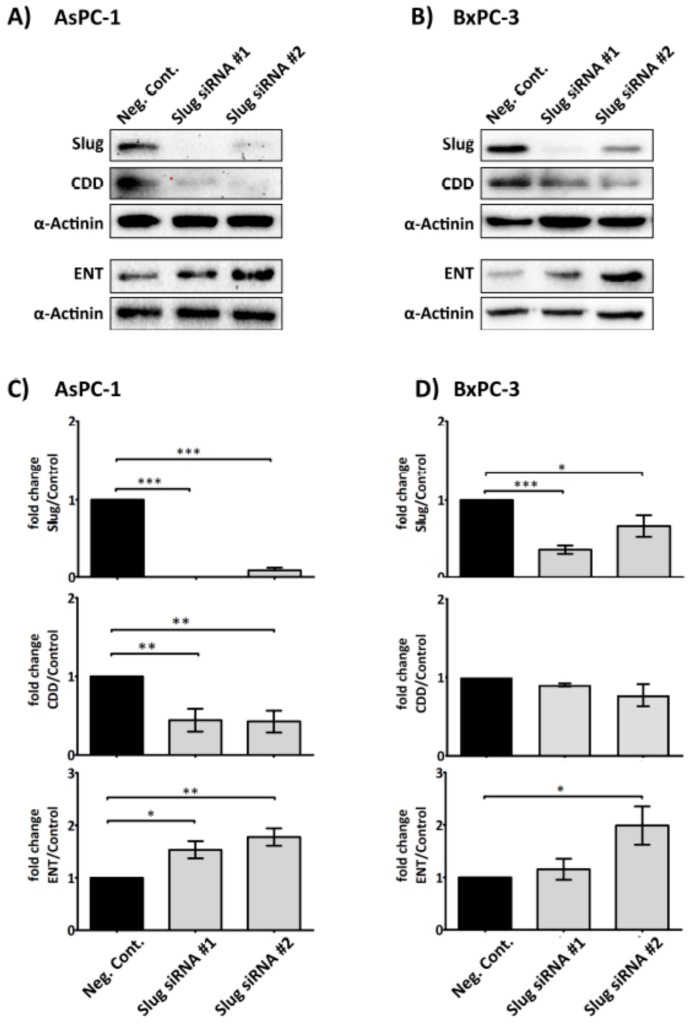
Slug mediates the expression level of CDD and hENT1 in PDAC. Representative immunoblots (**A**) and (**B**) show the expression of Slug, hENT1 and CDD in AsPC-1 and BxPC-3 after Slug-specific siRNA treatment. 3 × 10^5^ cancer cells were seeded in 6-well plate for 24 h. Slug siRNAs (the number #1 and #2 designate two different Slug siRNA sequences) or a negative control siRNA (75 nM) in serum/antibiotic-free media were added to the adherent cells and incubated for 24 h. The media was then replaced with fresh media (+10% fetal bovine serum (FBS)) and cells incubated for a further 24 h before cells were lysed and examined for the expression of the proteins of interest. Bar graphs (**C**) and (**D**) show the expression of Slug, hENT1, CDD and loading control α-actinin in AsPC-1 and BxPC-3 post siRNA treatment. The expression levels were quantified relative to the loading control. The columns represent the fold change of protein levels relative to the negative control siRNA treated cells (*n* ≥ 3). Data are presented as mean ± SEM. One way ANOVA with post-hoc Bonferroni’s Multiple Comparison Test was used to examine the significance of the mean *** *p* < 0.0001, ** *p* < 0.001, * *p* < 0.05. Neg. Cont.: negative control.

As platelet releasate was demonstrated to significantly increase Slug and alter the levels of CDD and hENT1 in PDAC cells, we next examined whether Slug is necessary for the modulation of CDD and hENT1 expression. A *SLUG* messenger RNA (mRNA) knockdown assay, using two different short interfering RNA (siRNA) sequences specific for *SLUG* mRNA and an irrelevant siRNA negative control, was performed to assess the changes in the expression of CDD and hENT1 in PDAC cells. Figure 4 clearly shows that the expression of CDD was significantly suppressed and, conversely, hENT1 was significantly increased in the absence of Slug in AsPC-1 cells. The protein expression change trends were also observed in the BxPC-3 cells, but the modulation was less pronounced and not statistically significant.

### 2.5. Platelet-Derived ADP and ATP Promote Slug and CDD Expression Levels in PDAC Cells

**Figure 5:**
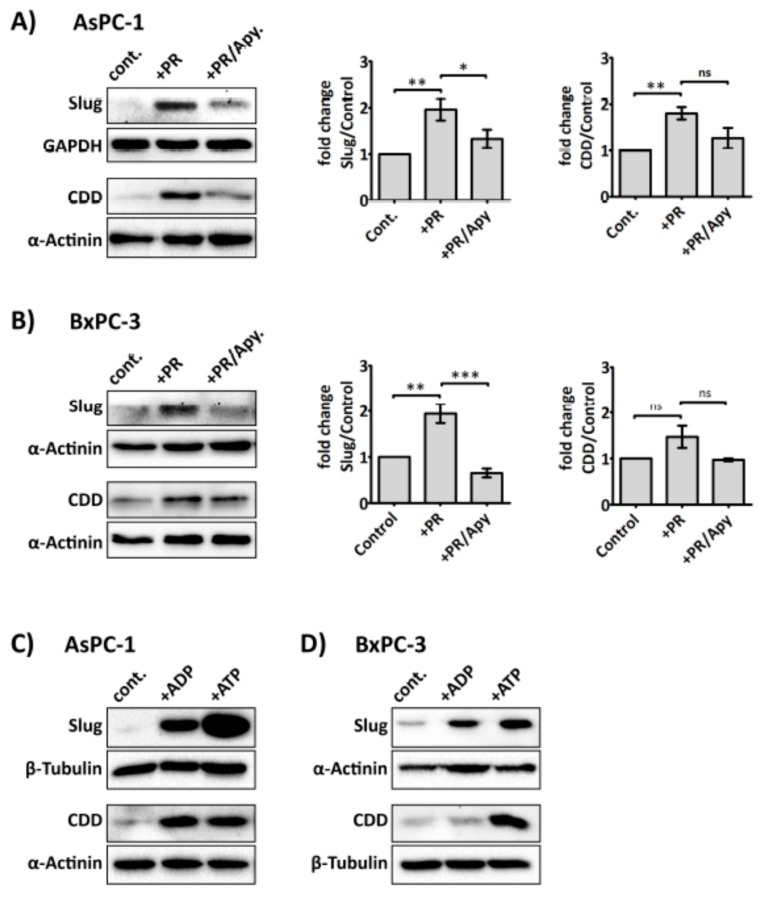
Platelet-derived ADP and ATP mediate Slug and CDD expression in PDAC cells. Representative immunoblots and bar graphs (**A**) and (**B**) show Slug and CDD expression in AsPC-1 and BxPC-3 cells after incubation with PR or PR pre-treated with apyrase (1 U/mL, for 30 min at 37 °C). 3 × 10^5^ cells were seeded in a 6-well plate for 24 h, then PR or apyrase pre-treated PR were added to the cancer cells for a further 24 h in serum-free media. The final concentration of PR used was equivalent to releasate from 5 × 10^8^ platelets/mL. Cell lysates were prepared and used in SDS-PAGE and immunoblotting as previously described. The expression levels of the proteins of interest were quantified relative to the loading control. The columns represent fold-change in protein expression level compared to control, vehicle-treated cells. Data are presented as mean ± SEM. One way ANOVA with post-hoc Bonferroni’s Multiple Comparison Test was used to examine the significance of the mean. *n* ≥ 4. *** *P* < 0.0001, ** *P* < 0.001, ** *P* < 0.05. Representative immunoblots (**C**) and (**D**), from two independent experiments, show Slug and CDD expression in AsPC-1 and BxPC-3 after exogenous ADP or ATP (100 µM) treatment for 24 h. cont.: control.

Several purinergic receptors are reported to be expressed on cancer cells, including PDAC cells, ((e.g., P2Y_1_ and P2X_7_, which are adenosine diphosphate (ADP) and adenosine triphosphate (ATP) receptor, respectively)) [38,39]. More interestingly, recent studies suggest that purinergic signalling can promote invasiveness and EMT in prostate cancer cells [40,41]. Since ADP and ATP are secreted from activated platelets, and our above data indicated that platelet-derived soluble factors were responsible for modulating Slug and CDD expression, we next investigated whether the enzyme apyrase—which catalyses the hydrolysis of ATP and ADP—could ameliorate the effect of platelet releasate on Slug and CDD expression in PDAC cells. Our results show that apyrase (1 U/mL) significantly negated PR-induced upregulation of Slug and CDD in cancer cells (Figure 5A,B) but apyrase did not significantly modulate hENT1 expression level (Supplementary Figure S3). Exogenous ADP and ATP (100 µM) also increased the expression levels of Slug and CDD in PDAC cells (Figure 5C,D). 

### 2.6. The Antiplatelet Drug, Ticagrelor, Reduces PR-Induced Akt, Erk Activation and Slug Upregulation in Cancer Cells

**Figure 6:**
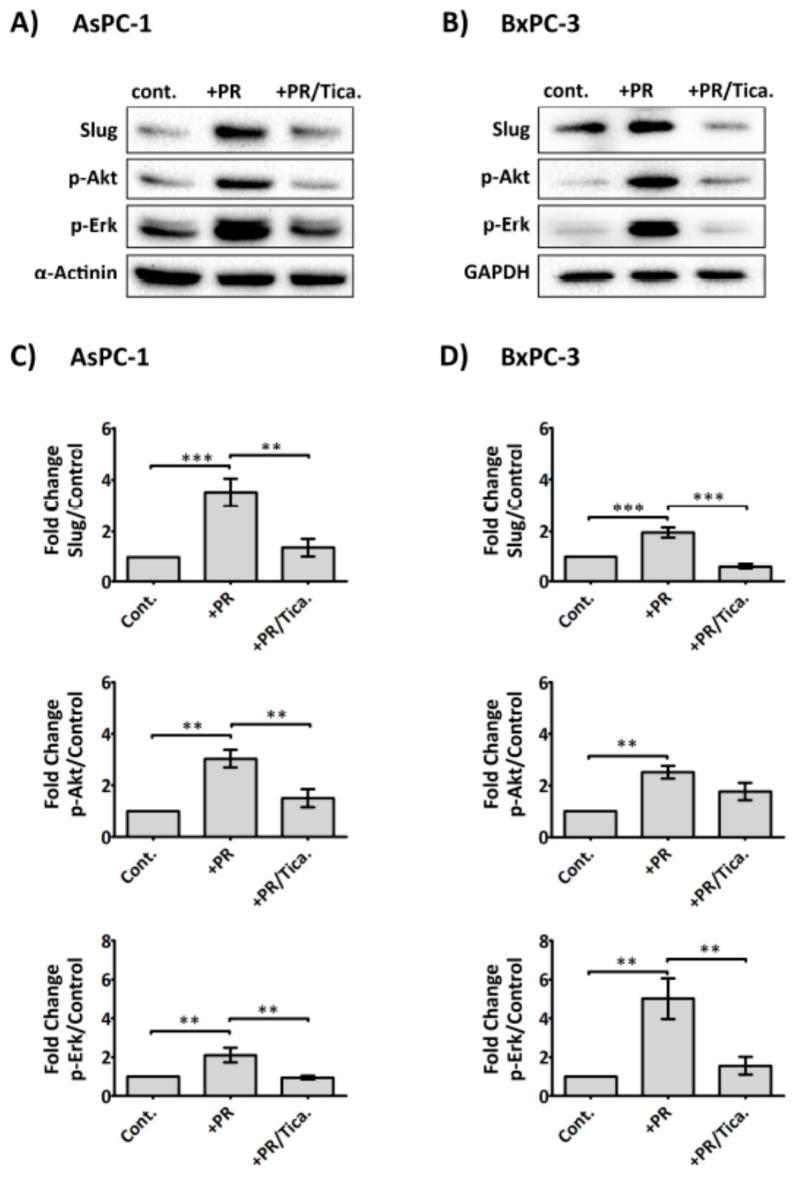
Anti-platelet drug ticagrelor, an antagonist of the purinergic receptor P2Y_12_ receptor, reduces the effects of PR on cancer cells. Representative immunoblots show Slug, p-Akt and p-Erk expression in AsPC-1 and BxPC-3 cells after incubation with PR ± ticagrelor (Tica, 10 µM) for 2 h. Sample preparations and immunoblotting were performed as previously described. The final concentration of PR used was equivalent to releasate from 5 × 10^8^ platelets/mL. Bar graphs (**C**) and (**D**) show the expression levels of the proteins of interest relative to the loading control. Columns represent fold change in protein expression level compared to control non-treated cells. *n* ≥ 5. *** *p* < 0.0001, ** *p* < 0.001, ** *p* < 0.05. Data are presented as mean ± SEM. One way ANOVA with post-hoc Bonferroni’s Multiple Comparison Test was used to examine the significance of the mean. cont.: control.

In view of the fact that platelet-derived ADP/ATP could significantly promote upregulation of Slug and CDD, two important markers of gemcitabine resistance, we, therefore, assessed whether AsPC-1 and BxPC-3 expressed the ADP receptor, P2Y_12_ by western blot analysis (Supplementary Figure S4), which confirmed its expression in both cell lines. We therefore assessed whether the clinically available P2Y_12_ receptor antagonist (ticagrelor) reduced PR-initiated signals in cancer cells. Cancer cells adherent in 6-well plates were treated with PR ± ticagrelor (10 µM) and incubated for 2 h in serum-free media. As shown in Figure 6, ticagrelor negated the PR effect on Akt, Erk activation and Slug upregulation in AsPC-1 and BxPC-3. Exogenous ADP and ATP (100 µM) were also able to induce Akt, Erk activation and Slug upregulation in cancer cells and these effects were completely blocked by ticagrelor (10 µM) (Supplementary Figure S5).

## 3. Discussion

Various types of cancer cells, including PDAC cells, can activate platelets, which in turn, can support tumour cell growth and metastasis [10,42,43,44]. Recent clinical studies have indicated an association between platelets and poor chemotherapeutic responses in cancer patients [45,46]. However, the mechanisms by which platelets confer chemoresistance to cancer cells have remained unclear. In accordance with several investigative studies on the impact of platelets on tumour growth [9,47,48], our data showed that platelet-derived factors could promote proliferation of AsPC-1 and BxPC-3 cancer cells and protected them from the cytotoxic effects of gemcitabine (Figure 1A,B). Previous studies indicated that cellular Akt or Erk activity was associated with gemcitabine resistance [49,50,51,52]. Here, after exposure to platelet releasate, a significant increase of Akt and Erk phosphorylation was detected in both cell lines, and the activation of these survival signalling molecules remained in the presence of gemcitabine (Figure 1C,D). Our results suggest that platelet releasate contained an active component (or components) capable of regulating Akt and Erk phosphorylation and promoting cell growth under chemotherapy. 

Platelet-derived TGF-β1 has been implicated as the major signalling regulator of cancer progression towards an invasive phase, through an induction of EMT [10,53,54]. Critical downstream effectors of TGF-β1 include receptor-regulated Smad2/3 [55,56,57] and Slug [58,59], a transcriptional repressor and regulator of EMT and chemoresistance [29,60]. In our study, the expression of Slug in both PDAC cell lines was significantly, and rapidly, augmented by the addition of platelet releasate (Figure 2A,B). However, the level of Slug did not lessen in the presence SB431542, a TGF-β1 receptor blocker. Furthermore, the phosphorylation of Smad2/3 by platelet releasate was inhibited by SB431542 in both cell lines, suggesting that the increase in Slug expression by PR in AsPC-1 and BxPC-3 was independent of TGF-β1R signalling (Figure 2C,D). The exposure to exogenous TGFβ1 showed that the cells may have distinct responses to the cytokine, as the upregulation of Slug expression was much more elevated in BxPC-3. Therefore, our data suggest that inhibition of platelet-derived TGF-β1 signalling was dispensable under these conditions and other factors apart from TGF-β1 could promote Slug expression. More importantly, when the cancer cells were grown in the presence of platelet-derived soluble factors and challenged with gemcitabine, Slug expression stayed elevated (Figure 2E,F). The signalling cascade through the complex Smad2/3/4 may be very important in some type of pancreatic cancer cells as Smad4 is a critical mediator of TGFβ signalling. However, Smad4 is also known to be inactivated in more than 50% of PDAC patients, and several in vitro studies have indicated that both AsPC1 and BxPC3 lack Smad4 protein expression [61,62,63,64,65], therefore its contribution to signal transduction is negligible in regards to these particular cancer cell lines.

The effectiveness of gemcitabine relies on the function of several proteins, including the influx protein, ENT1 [35], deoxycytidine kinase (dCK) (phosphorylates gemcitabine into an active form) [66], and CDD (deactivates gemcitabine) [36,37]. In our hands, we observed a statistically significant upregulation of CDD expression in cancer cells cultured with platelet releasate (Figure 3), suggesting that soluble factors from platelets could increase the capacity of cancer cells to deactivate gemcitabine. Inversely, the expression of hENT1 was decreased, at a statistically significant level in AsPC-1 and a trend reduction in BxPC-3. Reduced hENT1 implies that a lesser amount of gemcitabine could be transported inside the cells, thus increasing cell survival. Interestingly, degranulated platelets were also capable of instigating a significant upregulation of Slug in the cancer cells, suggesting a physical interaction between the surface molecules of platelets and cancer cells could induce intracellular signalling down the EMT pathway. Similarly, Labelle et al. have previously observed that a direct contact between cancer cells and platelets can activate a nuclear factor kappa-light-chain-enhancer of activated B cells, Nf-κB, pathway and EMT-like transformation in cancer cells independent of platelet-released factors [10]. Although studies indicate a common pathway for Nf-κB and Slug in regulating EMT [67,68], currently it is still unclear which receptors are involved in this cascade, and more studies are required to understand this interaction better.

The results of Slug knockdown indicated that the expression levels of CDD and hENT1 were dependent on Slug (Figure 4). Absent or reduction of Slug, especially in AsPC-1 cells, significantly reduced CDD and increased hENT1 expression, respectively. BxPC-3 Slug knockdown showed similar trends but were not statistically significant. This may be due in part to BxPC-3 being relatively more resistant to siRNA transfection. Our data highlight the novel role of Slug in the gemcitabine metabolism pathway and further validate its role in acquired-chemoresistance.

Since the increase of Slug expression by platelet releasate was not significantly dependent of platelet-derived TGF-β1, we hypothesised that Slug expression could be regulated by platelet-derived nucleotides, ADP and ATP. Platelets release ADP and ATP from their dense granules after interacting with stimuli such as cancer cells. Several studies have suggested nucleotides to be important in promoting tumour metastasis by increasing the permeability of the transendothelial barrier [43], maintaining tumour cell survival under duress [69,70], and eliciting EMT-related genes in cancer cells [40]. Here, we show that degradation of ATP and ADP by apyrase prevented the platelet releasate-induced increase of Slug and CDD expression in PDAC cells (Figure 5A,B). Furthermore, exogenously supplied ADP and ATP to AsPC-1 and BxPC-3 increased both the Slug and CDD expression level, suggesting a regulatory role for ADP and ATP in EMT and gemcitabine metabolism in cancer cells. 

Activation of purinergic receptors, including P2Y_1_ [71] and P2X_7_ [39,72], in PDAC cells have been shown to promote cancer cell growth. Here, we report a low-level expression of the ADP receptor P2Y_12_ in PDAC cell lines AsPC-1 and BxPC-3 (Supplementary Figure S4). This corroborated with the Human Protein Atlas data (https://www.proteinatlas.org/ENSG00000169313-P2RY12/pathology) which indicated elevated P2Y_12_ expression in pancreatic cancer tissue and negligible detection level in normal tissue [73,74]. P2Y_12_, considered the predominant receptor that mediates ADP-induced platelet activation [75] and detected primarily in platelets and brain tissues [76,77] has also been detected in other cell types including smooth muscle cells [78,79] and pancreatic islets [80]. Here, our data show that ticagrelor, an ADP receptor P2Y_12_ antagonist and clinically available antiplatelet drug, was effective at reducing platelet releasate-induced cancer cell activation (Figure 6). This was a direct effect of ticagrelor on the cancer cells as the cells were exposed only to platelet-derived soluble factors, not whole platelets. As ATP is an unstable molecule and can be rapidly hydrolysed to ADP and phosphate, the effects observed with exogenously administered ATP on the cancer cells may also be the results of the action of ADP. This is corroborated by the inhibitory effect of ticagrelor on cancer cells supplied with extracellular ATP (Supplementary Figure S5). It must be noted that the action of ticagrelor on the cell lines may also be due to its ability to weakly inhibit the uptake of adenosine by hENT1 [81,82], therefore preventing the pro-survival effect provided by adenosine uptake [83]. Furthermore, a drug review information (NDA number 22-433) from the U.S. Food and Drug Administration indicated that ticagrelor, at lower µM concentrations, can inhibit adenosine A3 receptor, and phosphodiesterase 5 (PDE5) [84]. Studies have shown that a blockage of A3 receptor reduced viability and chemotherapy resistance in glioblastoma stem-like cancer cells [85], whereas, inhibition of PDE5 potentiated gemcitabine in pancreatic cancer cells [86].

In summary, we show here for the first time that factors released from activated platelets can support PDAC cells to better survive under gemcitabine challenge by modulating CDD and hENT1 expression, both of which are controlled by Slug. The chemoresistance was mediated largely through platelet-derived nucleotides ADP and ATP. As tumours are exposed to high levels of extracellular nucleotides and nucleosides within the microenvironment [87,88,89], a supplementary strategy to target platelet activation and purinergic signalling in cancer treatment may be beneficial. Additionally, we showed a direct anti-cancer effect of ticagrelor, warranting a further assessment of the drug in the context of cancer therapy. 

## 4. Materials and Methods 

### 4.1. Reagents and Cell Lines

Apyrase, ADP, ATP, gemcitabine and ticagrelor were obtained from Sigma-Aldrich, (St. Louis, MO, USA). Inhibitor of the transforming growth factor-β (TGF-β) type I receptor, SB431542 was from Tocris Bioscience, Bristol, UK. AsPC-1 and BxPC-3 cell lines were obtained from ATCC and tested negative for mycoplasma (tissue culture facility routine testing). Cells were maintained in RPMI 1640 medium supplemented with 2 mM glutamine, 1 mM Sodium Pyruvate, 1 mM non-essential amino acids (all from Gibco^®^ Life Technologies Australia Pty Ltd., Mulgrave, Australia), and 10% Foetal Bovine Serum (FBS, from Bovogen Biologicals, Keilor East, Australia).

### 4.2. Preparation of Human Washed Platelets

Blood from healthy volunteers was drawn into ACD (acid-citrate-dextrose—15% *v*/*v*) with informed consent in concordance with the Curtin University Human Research Ethics Committee (approval number HR54/2014). Washed platelets were prepared as previously described [90]. Platelet count was adjusted to 5 × 10^9^/mL and suspended in HEPES-Tyrode’s buffer (5 mM HEPES, 5.5 mM glucose, 138 mM NaCl, 12 mM NaHCO_3_, 0.49 mM MgCl_2_, 2.6 mM KCL, 0.36 mM NaH_2_PO_4_, 1.8 mM CaCl_2_, pH 7.4). To prepare degranulated platelets (DG Plt) and platelet releasate (PR), washed platelets were activated with cross-linked CRP (collagen-related peptide—1 µg/mL, Auspep, Tullamarine, Australia) at 37 °C for 30 min. The DG Plt pellet was separated from the PR supernatant by centrifugation at 5000 *g* for 10 min. 

### 4.3. Cell Viability 

Cancer cells were seeded at 5000 cells/well in a 96-well plate. After 24 h, the medium was replaced with serum-free RPMI 1640 medium supplemented with platelet releasate and gemcitabine (0.1 to 100 µM) and incubated for a further 72 h. Cell viability was measured by detecting the metabolic activity of live cells using Alamar blue (Resazurin sodium salt, Sigma-Aldrich, St. Louis, MO, USA) as previously described [91]. Briefly, 10 × Alamar blue was added as 10% of the sample volume; then the plate was incubated for 1 h (AsPC-1) or 2 h (BxPC-3) at 37 °C. Alamar blue reagent is a non–toxic cell health indicator that is converted to fluorescent red colour in living cells, and the fluorescence (ex/em 570 nm/610 nm) was quantified by a plate reader (EnSpire Multimode, PerkinElmer^®^, Waltham, MA, USA). 

### 4.4. Western Blot:

The level of protein expression was examined by Western blot. Specific antibodies against Slug, p-Akt (Ser473), p-Smad2 (Ser465/467)/Smad3 (Ser423/425), p-Erk1/2(Thr202/Tyr204), β-tubulin, α-Actinin and Glyceraldehyde 3-phosphate dehydrogenase (GADPH) were obtained from Cell Signaling Technology^®^ (Danvers, MA, USA). Rabbit anti-CDD and hENT1 antibodies were obtained from Santa Cruz Biotechnology (Dallas, TX, USA). Rabbit P_2_Y_12_ [EPR18611] monoclonal antibody was obtained from Abcam Biotechnology (Cambridge, UK). Tumour cells were seeded in a 6-well plate. After 24 h, cells were serum starved for 6 h, then subjected to different treatments, and the plate was incubated for the specified time. Cells were then washed 3 times with ice-cold Tris Buffered Saline (TBS, 137 mM, sodium chloride, 20 mM Tris, pH 7.6) and lysed in ice-cold cell extraction RIPA buffer (ThermoFisher Scientific Inc., Waltham, MA, USA) supplemented with Protease/Phosphatase Inhibitor Cocktail (Cell Signaling Technology^®^, Danvers, MA, USA). Forty-five µg of cell lysate was then analyzed by sodium dodecyl sulphate–polyacrylamide gel electrophoresis and immunoblotted for the relevant protein. Equal loading was verified by immunoblotting for the house-keeping proteins (β-tubulin, α-actinin or GADPH). 

### 4.5. Slug Knockdown

Cancer cells were seeded at 3 × 10^5^ cells/well in a 6-well plate and maintained for 24 h. Slug siRNAs (two different Slug-specific siRNA sequences) or a negative control siRNA (75 nM), prepared using DharmaFECT1 transfection agent (GE Dharmacon, Lafayette, CO, USA) in serum/antibiotic-free media, were added to the adherent cells and incubated for 24 h. The two Slug-specific siRNAs GAAUGUCUCUCCUGCACAA (#1) and UCUCUCCUCUUUCCGGAUA (#2) were purchased from GE Dharmacon, Lafayette, CO, USA. The Silencer^®^ negative control (sequence: proprietary, designed to have no significant similarity to human sequences, catalogue #AM4635) was from Ambion^®^, (ThermoFisher Scientific Inc., Waltham, MA, USA). The media (containing the transfection mixture) was then replaced with fresh media (RPMI + 10% FBS), and the plate was further incubated for 24 h. After washing cells with TBS, the cells were then lysed with RIPA buffer, and western blots used to examine protein levels of expression. It must be noted that the BxPC-3 cell line was relatively difficult to transfect, likely due to the expression of extracellular DNA on the cell surface [91], and to achieve efficient transfer of siRNAs, the cells were treated with DNase (60 U/mL, STEMCELL Technologies, Vancouver, BC, Canada) for 3 h, then washed 3 times with EDTA (50 mM), before addition of the transfection mixture as described above.

### 4.6. Statistical Analysis

Data were analysed using GraphPad PRISM 5.0 software (GraphPad Software, Inc, CA, USA). Results are expressed as the mean ± standard error (SEM). One-way ANOVA with posthoc Bonferroni’s Multiple Comparison Test was used to examine the significance of the mean. Differences were considered significant at *p*-value less than 0.05.

## 5. Conclusions

Our results report for the first time that platelets can regulate the expression of markers of gemcitabine resistance in pancreatic cancer cells, hENT1 and CDD. Slug, a mesenchymal transcription factor and mediator of EMT processes, was shown to regulate the expression of both hENT1 and CDD. Furthermore, we showed that platelet-derived ADP and ATP could modulate the level of Slug and CDD, and activated survival signals in cancer cells. Finally, ticagrelor, a P2Y_12_ inhibitor, could act directly on the cancer cells and abrogated the survival signals initiated in cancer cells by platelet-derived ADP and ATP. Future studies are required to examine whether targeting platelet activation and purinergic signalling in cancer cell could reduce chemotherapy resistance and cancer metastasis.

## Supplementary Materials

The following are available online at http://www.mdpi.com/2072-6694/9/10/142/s1.
