# Supplementary material for: The Role of Platelet-Derived ADP and ATP in Promoting Pancreatic Cancer Cell Survival and Gemcitabine Resistance
Source: Cancers (Basel). 2017 Oct 24;9(10):142. doi: 10.3390/cancers9100142 (PMC5664081; doi:10.3390/cancers9100142)
Supplement: Supplementary file 1 [file cancers-09-00142-s001.docx]

**Supplementary Materials: The Role of Platelet-Derived ADP and ATP in Promoting Pancreatic Cancer Cell Survival and Gemcitabine Resistance**

Omar Elaskalani, Marco Falasca, Niamh Moran, Michael C. Berndt and Pat Metharom *


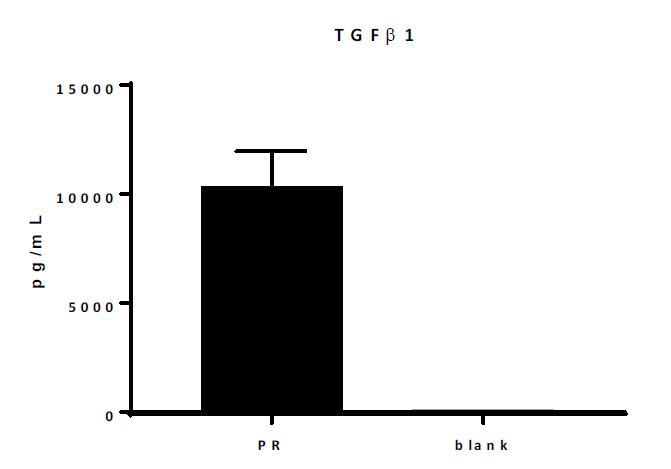


**Figure S1.** TGF-β1 in platelet releasate.PR from four different donors were prepared at 5 × 108platelets/ml and were analysed for the presence of human TGF-β1with an ELISA kit (Biosensis, Pty Ltd, CA, USA). *n* = 4, mean = 10360 ± 3195 pg/ml.

**Figure S2.** TGF-β1 upregulates Slug in BxPC-3 but not in AsPC-1. Cancer cells were treated with platelet releasate (PR), ADP (100 µM), ATP (100 µM), and TGF-β1 (10 ng/mL) for 2 h. PR, ADP and ATP induced Slug upregulation in both cell lines. TGFβ1 activated SMAD2/3 in both cell lines, however, highly upregulated Slug in BxPC-3, but not AsPC-1. * Each blot is a representative sample of 3 independent experiments with similar results.


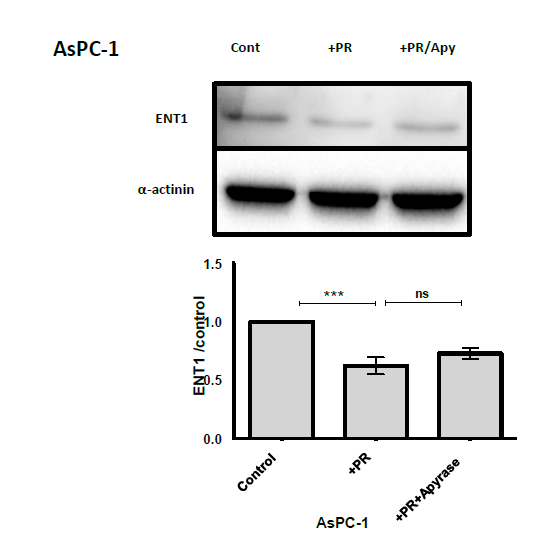


**Figure S3.** Apyrase did not significantly alter hENT1 expression in PR-treated cancer cells. Representative immunoblots and bar graphs show hENT1 expression in AsPC-1 cells after incubation with PR or PR pre-treated with apyrase (1 U/mL, for 30 min at 37 °C). 3 × 105cells were seeded in a 6-well plate for 24 h, then PR or apyrase pre-treated PR were added to the cancer cells for a further 24 h in serum-free media. The final concentration of PR used was equivalent to releasatefrom 5 × 108platelets/mL.Cell lysates were prepared and used in SDS-PAGE and immunoblotting as previously described. The expression levels of the hENT1 were quantified relative to actinin.One way ANOVA with post-hoc Bonferroni’s Multiple Comparison Test was used to examine the significance of the mean. *n* = 4. *** *p* < 0.0001.


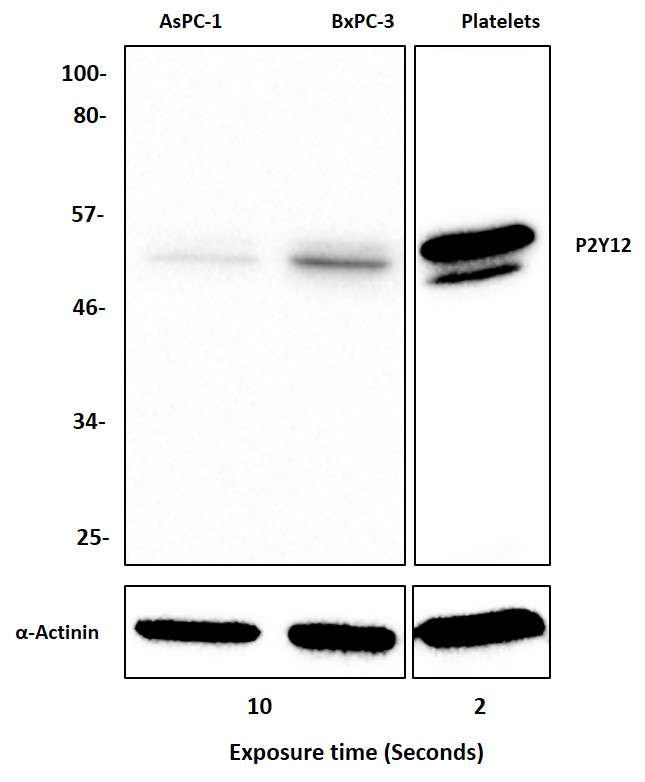


**Figure S4.** Expression of P2Y12 in PDAC cell lines AsPC-1 and BxPC-3. Cell lysates from AsPC-1, BxPC-1 and platelets were prepared using RIPA lysis buffer supplemented with protease inhibitor cocktail (Cell Signaling Technology). 20 µg of cell lysate with Laemmli sample buffer was loaded per lane, and immunoblotting was performed as mentioned in methods and materials. Rabbit anti-P2Y12 [EPR18611] monoclonal antibody, from Abcam Biotechnology Company was used to detect P2Y12 and, after membrane stripping, equal loading was verified using the house keeping protein α-Actinin (Cell Signaling Technology). P2Y12 is highly expressed in platelets, therefore the development of the membrane was performed using signal accumulation mode (ChemiDoc, Biorad Imaging system), and the bands in PaCa cells and platelets are shown at different exposure time.


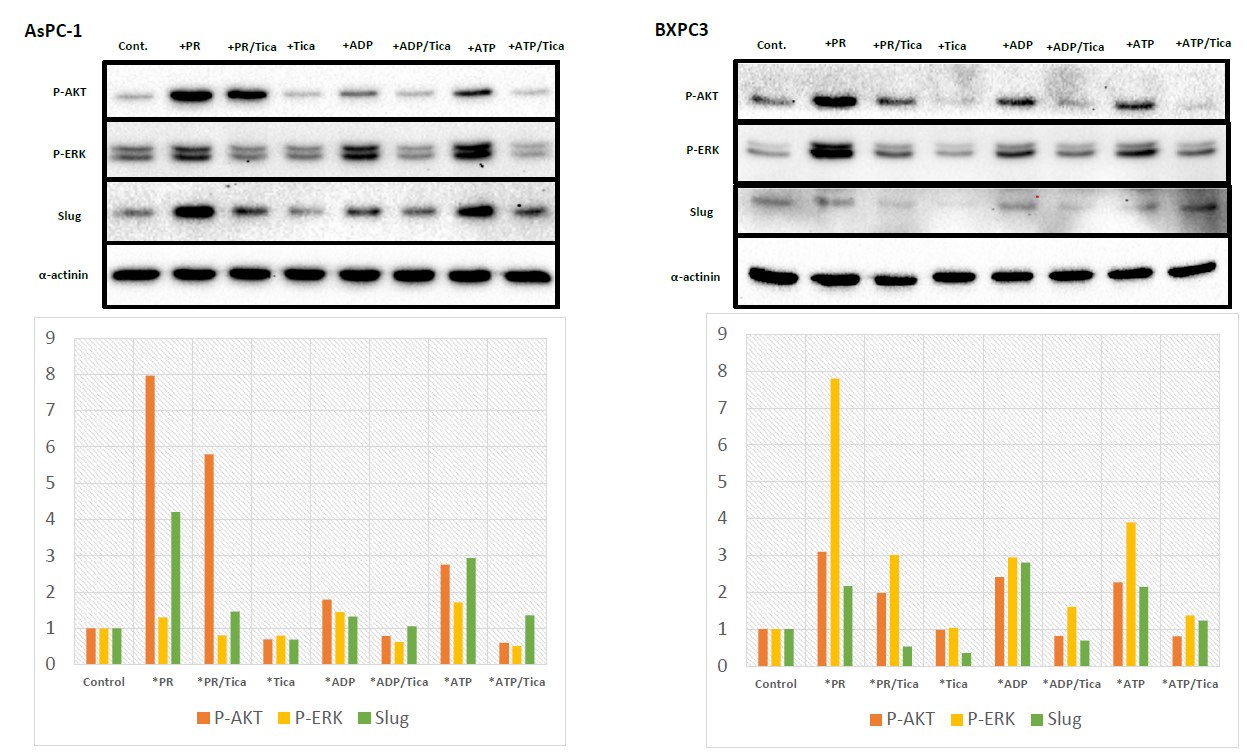


**Figure S5.** Ticagrelor inhibits the effects of ADP and ATP in the cancer cells. Cancer cells were treated with platelet releasate (PR), ADP (100µM) and ATP (100 µM) ± ticagrelor (10 µM) for 2 h. ticagrelor (10 µM) reduced PR, ADP and ATP-induced p-Akt, p-Erk and Slug upregulation, possibly by the blocking purinergic receptor P2Y_12_ that is found to be expressed on both AsPC-1 and BxPC-3. ATP is an unstable molecule, and its effects can also include the effects of its hydrolysed product, ADP. * Each blot is a representative sample of 2 independent experiments with similar results.
